# Photonic Low Cost Micro-Sensor for in-Line Wear Particle Detection in Flowing Lube Oils

**DOI:** 10.3390/s17030586

**Published:** 2017-03-14

**Authors:** Jon Mabe, Joseba Zubia, Eneko Gorritxategi

**Affiliations:** 1 IK4-Tekniker, Eibar 20600, Spain; 2 Department of Communications Engineering, ETSI de Bilbao, University of the Basque Country (UPV/EHU), Alameda de Urquijo s/n, Bilbao 48013, Spain; joseba.zubia@ehu.es; 3 Advanced Monitoring Technologies, Eibar 20600, Spain; egorritxategi@atten2.com

**Keywords:** optoelectronic and photonic sensors, object recognition, optical sensors, optical microscopy, optical diffraction, lubricating oils, hydraulic fluids, maintenance

## Abstract

The presence of microscopic particles in suspension in industrial fluids is often an early warning of latent or imminent failures in the equipment or processes where they are being used. This manuscript describes work undertaken to integrate different photonic principles with a micro- mechanical fluidic structure and an embedded processor to develop a fully autonomous wear debris sensor for in-line monitoring of industrial fluids. Lens-less microscopy, stroboscopic illumination, a CMOS imager and embedded machine vision technologies have been merged to develop a sensor solution that is able to detect and quantify the number and size of micrometric particles suspended in a continuous flow of a fluid. A laboratory test-bench has been arranged for setting up the configuration of the optical components targeting a static oil sample and then a sensor prototype has been developed for migrating the measurement principles to real conditions in terms of operating pressure and flow rate of the oil. Imaging performance is quantified using micro calibrated samples, as well as by measuring real used lubricated oils. Sampling a large fluid volume with a decent 2D spatial resolution, this photonic micro sensor offers a powerful tool at very low cost and compacted size for in-line wear debris monitoring.

## 1. Introduction

**Figure 1:**
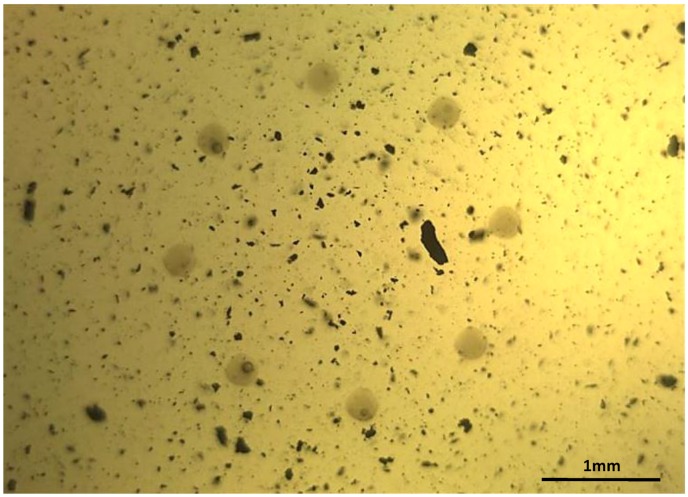
Image of a real lubricant sample from a gearbox in a wind mill, where large and small particles can be observed suspended in the fluid. The presence of this wear was evidencing a failure.

The presence of solid particles in suspension in industrial fluids, such as lubricants or hydraulic fluids, is frequently an early warning of latent or imminent faults in the machines or processes where they are being used [1,2], as it can be seen in Figure 1, where the contaminated lubricant of a gearbox about to fail is displayed. Therefore, the early detection of the presence of these wear particles is a key objective in a proper predictive maintenance program, complementing other machine condition monitoring approaches (CMS) such as vibration analysis [3]. The laboratory analysis of oil samples looking for evidence of wear was first introduced in 1948 [4], and this traditional off-line fluid analysis is still an important asset for several maintenance programs, especially when high accuracy and low detection limits are needed. However, the number of in-line sensor solutions is increasing steadily in the last decades [5,6,7,8]. The reason behind this growing number of in-line fluid monitoring technologies is found in a clear market request for: (i) real time results for critical infrastructure monitoring (gas turbines, aircraft engines) where the latency of the off-line laboratory tests is not acceptable [9,10]; (ii) solutions for applications with challenging and expensive off-line sample acquisition (e.g., off-shore wind turbines) [11,12]; and (iii) solutions for closing the control loops, as for example in torque regulation in run-in phases of gears or drive trains manufacturing [13]. These use cases are clear examples demanding a tradeoff solution between an immediate availability of information and a reduction in the accuracy of the results, which is the ideal scenario for solutions like the one presented in this paper.

### 1.1. Wear Debris Sensors

Considering the features normally required of a wear debris monitoring sensor (asides the chemical and physical fluid compatibility), the following items can be highlighted [14,15]:

1. Output of normalized and calibrated data as specified by relevant standards of fluid cleanliness levels like ISO 4406, NAS 1638 or SAE AS4059, that basically summarize the particle count per mL classified in different size ranges (e.g., >4 μm, >6 μm, >14 μm)
2. Achieve a minimum detection limit of approximately 2 μm in particle size, which is directly proportional to the ability of the sensor to detect changes in contamination levels earlier.
3. Offer sensibility to metallic and non-metallic particles, allowing the sensor to detect either endogenous debris (e.g., iron or steel particles from mechanical parts, debris from seals) or exogenous contamination such as silicon dust, water or air.
4. Maximize sampling volume, while minimizing the measurement latency, which is proportional to mL analyzed per unit of time and determines the significance of the measurement.
5. Classify particles in groups (e.g., cutting, sliding or fatigue) for root cause analysis of the faults, which is normally determined by the particle shape and size.
6. Operate reliably and offer accurate measurements in conditions of turbidity, opacity and water and air bubble presence in fluid samples.
7. Offer a competitive total cost of operation (price, installation and maintenance of the sensor) compared to the off-line laboratory tests.

Regarding the measurement principles, there are two predominant technologies for in-line wear debris sensors: magnetic or inductive detectors and photonic detectors [16,17]. While the magnetic or inductive solutions offer a very good performance for high flow rates, allowing them to monitor large fluid volumes in real time, they suffer some drawbacks in terms of resolution and minimum detectable particle size, and they are affected by electrical noise and vibrations [18]. Additionally, they are not able to detect non-metallic particles, and shape recognition is not an option either [19].

**Figure 2:**
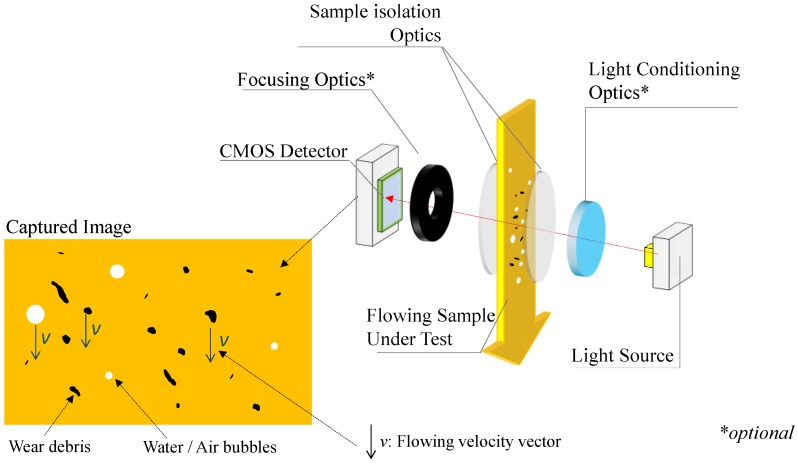
Block diagram depicting a direct imaging wear debris sensor analyzing a flowing lubricant.

On the other hand, photonic-based particle counters can be found, with different measurement principles available such as light scattering, light obscuration, or direct imaging [20,21]. However, among the different alternatives, the direct imaging systems (described in the Figure 2) are efficient solutions when the target for the minimum particle size is above 1 or 2 μm, preferably 4 μm, as it is the case for the ISO 4406 standardized applications, which are the most common ones for in-situ systems. The direct imaging systems offer a good ferrous and non-ferrous particle detection sensibility, allow shape recognition, and normally these solutions are relatively reliable in the presence of air and water bubbles [22]. However, these systems are normally constrained to very low flow applications and normally require a dedicated pumping system for bypassing the sample to the sensor in a regulated flow rate.

In addition, due to the physical limits of the focusing optics needed for Direct Imaging, these solutions are not able to measure large samples of fluids. The limitations of traditional microscopes straightly affect in the direct imaging solutions, because, if high resolution is required (e.g., 4 μm) the field of view (FOV) and depth of field (DOF) are constrained, which aggravates the problem of probing large sample volumes in real time. The situation gets even worse if relatively simple lens solutions are required (instead of bulky tele-centric optics for instance) due to restrictions in the cost and dimensions of the final system. As a reference for the reader, a sensor system mounting a lens with an effective focal length of 9.6, and a CMOS of 10 M Pix is able to measure approximately 0.025 mL (FOV 36 mm^2^/DOF 250 μm) of fluid per each sampling, achieving a resolution of around 3 μm [23]. These major limitations in resolution, FOV and DOF of traditional lens-based direct imaging sensors pave the way for applying lens-less microscopy techniques to wear debris monitoring. Lens-less microscopy is an emerging cost-effective technique that enables capturing high resolution images of 1 μm or below, over large FOV and long DOF with simple, compact and lightweight settings [24,25].

### 1.2. Lens-Less Imaging

Formulated in the late 1940s by the Hungarian engineer Dennis Gabor, the principle of lens-less imaging describes a new way for getting a perfect image. He proposed to capture an image of the interference pattern between the light that illuminates an object and the light diffracted by that object. Thanks to the amplitude and phase information, the collected light pattern contains all the information to reconstruct an image of the object [26]. Therefore, a lens-less imaging system only requires a light source, an aperture (pinhole) for generating the diffraction pattern, and an image sensor for capturing this light pattern. 

Based on these theoretical principles, the lens-less imaging or microscopy was later developed in the 1960s [27], and it has been evolving since then. Recently, mainly driven by technological developments in CMOS cameras, light sources and data processing resources, there has been an increasing number of applications and published works in the field of lens-less microscopy, but these have been mainly oriented to biological applications, focusing on the inspection of microorganisms and cells in biological samples [28]. 

**Figure 3:**
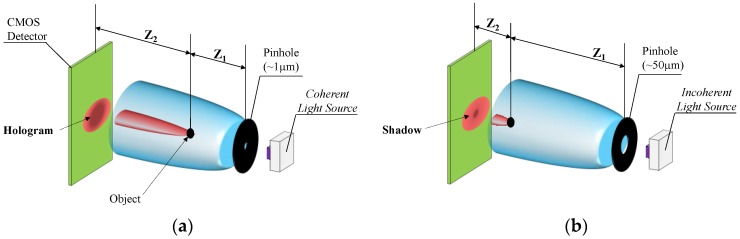
Coherent (**a**) and Incoherent (**b**) lens-less approaches.

There are different configurations for constructing a lens-less imaging system, basically depending on the properties of the light source, their detection/imaging geometries and reconstruction or image-processing algorithms (e.g., FFT based original object reconstructions or direct use of the interference patterns). Each configuration is able to generate images of focused objects situated between the light source and the image sensor, but the details of these captured images will differ. With several particularizations, we found two main categories, Digital In-line Holography (DIH) using coherent light and Incoherent Lens-Less On-Chip imaging [29] (see Figure 3).

The coherent lens-less option requires integrating a coherent light source, such as a LASER, with a pin hole with an aperture diameter of approximately 1 μm. This configuration assures the temporal and spatial coherence of the light hitting the sample object, enabling a later reconstruction of the captured holographic images into the original objects with a resolution below the micrometer. This configuration requires to set a distance in the range of some millimeters (~1000 · λ) between the object and the aperture (Z_1_), whereas the distance between the CMOS sensor and the object (Z_2_) is about some centimeters, therefore Z_2_ ~ 10 × Z_1_.

The incoherent lighting approach represents the simplest alternative within the lens-less arena because it avoids the need for advanced optical components like lasers, replacing them with standard light sources. In this configuration, the detector receives a self-interfering diffraction pattern generated when the light beam hits the target object. This pattern could be understood as the shadow generated by the object blocking the incoming light to the detector and could be directly used for the object’s size recognition, avoiding the use of computationally intensive holographic reconstruction algorithms [30,31]. In this case, the target objects should be located right above the detector, minimizing Z_2_ distance to 1 or 2 mm, and the distance from the objects to the pinhole should meet Z_1_ >> Z_2_ relation. Additionally, in the incoherent mode, the aperture diameter is normally above 50 µm.

One of the best advantages of the incoherent mode is its suitability for dense samples (samples with a lot of particles) because the cross-interference in the diffraction patterns of the different adjacent particles is near zero, whereas it is an important factor for the coherent version [32].

It becomes evident that the potential benefits of the lens-less imaging in terms of resolution, FOV, DOF and simplicity of the solution described in the literature, would positively contribute in the field on the in-line/on-line wear particle counting and classification. Even in the simplest expression of the lens-less microscopy, the incoherent mode, the solution looks promising due to a relatively large sample volume monitoring (FOV ~20 mm^2^, DOF ~1 mm), resolutions of approximately 2 μm, simplicity and robustness of the hardware setup, small size of 50 mm × 50 mm × 10 mm allowing the integration of the solution on larger structures [33,34], and last but not least, the possibility of avoiding the holographic reconstruction algorithms (which take several seconds in a PC station with a graphical processing unit) and working directly with the ‘spatial signatures’ or shadows generated by the light absorbed by particles in the sample volume.

An application of a lens-less approach for solving the detection of wear particles is presented in [35], however, it only dealt with static fluids and requires some external hydraulic conditioning systems, such as electro-valves or flow controllers, to stall the fluid while the images of the sample are taken. The need of these bulky hydraulic components jeopardize the low cost and compact size potential of the lens-less approach. However the system evolution for dealing with non-static fluid conditions is not straightforward, and several modifications especially in the lighting and image capture system, are required as described in the next section. 

### 1.3. Precision Imaging of Moving Objects

The current sensor proposal addresses the problem of the low sampling volume from a twofold approach. It is evident that the enhancements brought by the lens-less technology in terms of higher FOV, DOF and resolutions are contributing to acquire a sharp image of a larger sample volume. However, as mentioned above, the direct application of the lens-less solution still requires to regulate or stop the flow of the sample crossing the acquisition area.

**Figure 4:**
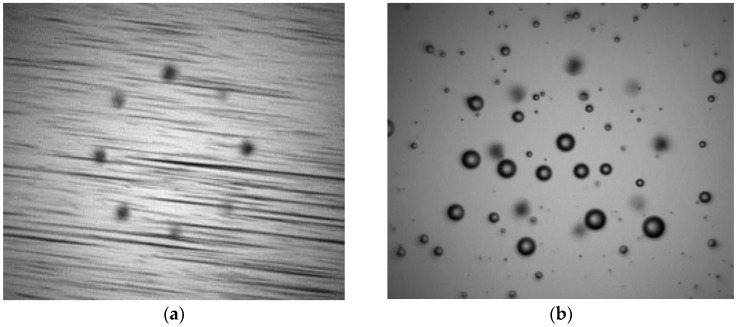
Example of distorted image due to the Rolling Shutter effect recording moving objects suspended in a fluid (**a**). Same objects recorded using synchronized stroboscopic illumination (**b**) generating a non-distorted image. (**a**) image has been recorded using an exposure time of 23 ms and a flash gain of ×1 with a duration equal or longer than the exposure time. The (**b**) image instead uses a flash pulse of 4 µs to allow the smear-free images but requires applying a flash gain of ×40 to achieve a similar image luminance level. In the (**b**) case, the effective exposure time is limited by the flash pulse duration, regardless of the configured pixel exposure.

The problem is related with the distortion or blur occurring at images capturing objects moving at relatively high velocities (see Figure 4). This distortion is caused because the target objects displaces its position within the acquisition area faster than the pixel acquisition time, being this acquisition divided in the pixel exposure time and on the pixel read-out time. This is widespread problem in the machine vision and imaging fields, and applications such as traffic management, metrology and robotics inspection all need to image fast-moving objects without smear or distortion. Techniques such as Global Shutter Imagers or Global Reset Rolling Shutter CMOS systems have risen as candidates to meet the requirement of capturing smear-free images of fast moving objects [35,36]. Additionally, there are some software based approaches to restore motion blurred particle images [37] applied to the in-line wear debris monitoring, however, they have only been validated at low flow rates of 1 mL/min and large particles.

The solution implemented in the current sensor proposal uses a stroboscopic light control (high amplitude pulsed light) system emulating a Global Shutter CMOS mode using a Rolling Shutter CMOS (with global pixel reset feature enabled). The CMOS frame capture trigger is synchronized with the LED light switching on, allowing the acquisition of semi-static images of moving objects avoiding the Jello effect or the diagonal bending of images, characteristic of rolling shutter captures of fast moving objects. Indeed, a Global Shutter CMOS could have been chosen for enabling non-distorted images, but the cost and size of Global Shutter sensors against the Rolling Shutter ones is much higher, especially in high resolution versions [38]. 

**Figure 5:**
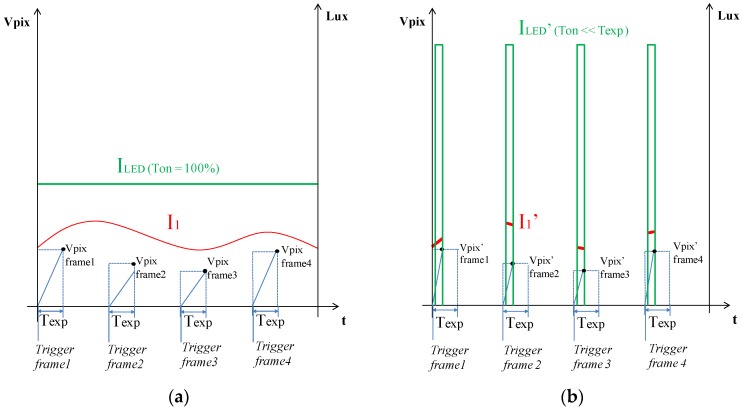
Graphical example of the irradiance received by the CMOS sensor (V_PIX_ = $R\left(\frac{V}{\mathrm{Lux}\cdot s}\right)$·I_1_(Lux)·T(s)) on each frame on a continuously illuminated case (**a**) and on the stroboscopic case (**b**). I_LED_ and I_LED_’ represents the light power generated at the emitter, whereas I_1_ and I_1_’ are the light remaining after crossing the whole system (optics, sample, etc.) and getting to the CMOS surface. Indeed, I_1_ and I_1_’ are proportional to the light emitted by the sources, I_LED_ and I_LED_’. T_EXP_ represents the exposure time configured at the CMOS, being the amount of time that each pixel is integrating the light received with the given sensor responsivity $R\left(\frac{V}{\mathrm{Lux}\cdot s}\right)$. Additionally, T_ON_ describes the light pulse duration, being 100% of time for the (**a**) case and T_ON_<<T_EXP_ for the (**b**) configuration. Note that the pulse start is synchronized with the frame triggering.

The stroboscopic-based illumination and capture system enables working with higher flow rates as the flash pulse duration is reduced, provided that enough light power reaches the detector for the later processing of the images. However, reducing the pulse length drastically impacts the effective amount of light available, which is especially important when illuminating opaque fluid samples. From Figure 5, it can be concluded that, if the design goal is to maintain the same light level at the CMOS detector (V_PIX_’ ~V_PIX_), then the emitted light power should meet I_LED_’ >> I_LED_ as described in Equations (1) to (4) (these calculations are further discussed in later sections). As the maximum light power amplitude (I_0_’) is limited by the technology, the optimal pulse duration (T_ON_) for each sensor configuration is a trade-off between the maximum flow rate and the compatibility with darker fluid samples:

(1) $$I_{1}\propto I_{\mathrm{LED}}\;,I_{1}\prime \propto I_{\mathrm{LED}}\prime$$

(2) $$V_{\mathrm{pix}}=R\left(\frac{V}{\mathrm{Lux}\cdot s}\right)\times I_{1}\left(\mathrm{Lux}\right)\times T_{\mathrm{EXP}}\left(s\right)$$

(3) $$V_{\mathrm{pix}}\prime =R\left(\frac{V}{\mathrm{Lux}\cdot s}\right)\times I_{1}\prime \left(\mathrm{Lux}\right)\times T_{\mathrm{ON}}\left(s\right)$$

(4) $$T_{\mathrm{EXP}}\gg {\;T}_{\mathrm{ON}}\to I_{1}\prime \gg I_{1}\;\to V_{\mathrm{pix}}\prime \;\sim {\;V}_{\mathrm{pix}}$$

Additionally, this light shortage is aggravated due to inclusion of the pinhole requested by the lens-less configuration, which acts as a spatial filter blocking an important amount of the light emitted by the source.

Therefore, bringing all the potential advantages of the lens-less optics and stroboscopic illumination system to an integrated sensor is not straightforward, due to some application-specific requirements that need to be met as the mentioned maximum sample flow rate or the maximum pressure to be born by the sample cell. The target operating conditions for the lubricant sensors may require standing working or burst pressures above 10 bars, requiring a protective mean between the running sample and the CMOS detector that will directly impact on the possibility of reducing the lens-less Z_2_ distance.

The following sections describe the work performed for the application of the aforementioned photonic principles into an optical microsensor for the detection of wear particles in running industrial fluids with clear maximum pressure and flow rate specifications.

## 2. Materials and Methods

This section describes the different test benches and prototypes developed with the objective of validating the proposed sensor approach that enables the largest FOV, DOF, highest resolution and maximum compatible flow rate to fulfill the industrial requirements for particle counters. The development starts with a theoretical approach for describing the lens-less setup, and a proof-of-concept validation phase in an optical test bench. Some theoretical approximations follow, defining the light power budget in relation with the maximum compatible fluid flowing velocity, and calculating the different structural constraints due to maximum fluid pressure. Some intermediate results are displayed along the section, which verify the different modules later included in the sensor.

### 2.1. Optical Test Bench

As a first step in the proof-of-concept realization, a custom optical test bench has been arranged. The main objectives of this setup are: (i) definition of the Z_1_ and Z_2_ distances, (ii) definition of the pinhole diameter D; (iii) characterization of system performance in terms of resolution, FOV and DOF; and (iv) characterization of the stroboscopic light for enabling the maximum compatible sample flowing rate.

**Figure 6:**
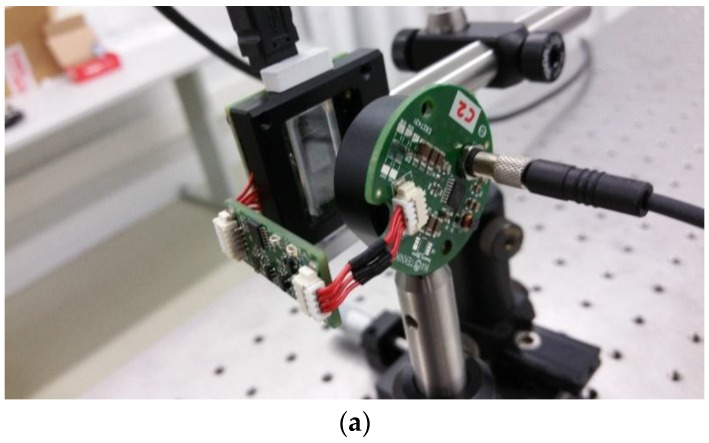

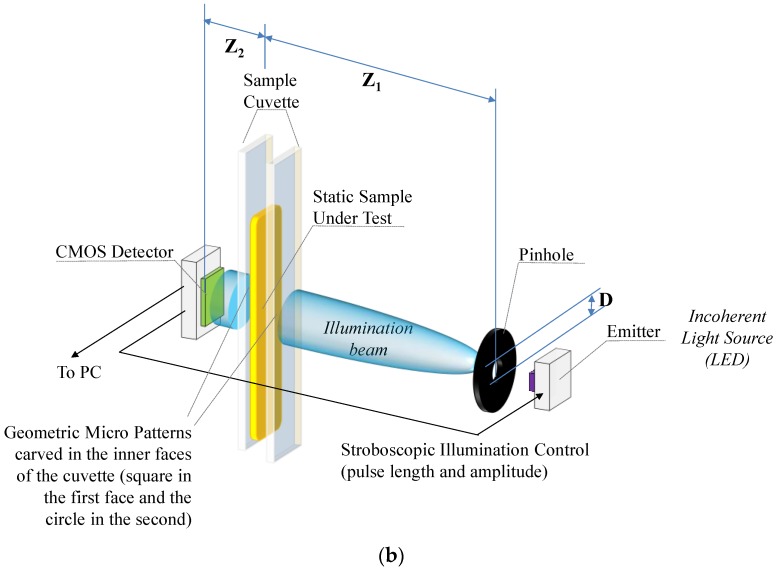
(**a**) Photo and (**b**) block diagram of the optical test bench.

The photonic test bench (see Figure 6) comprises a customized light source based on an array of XLamp white LEDs from Cree (Durham, NC, USA) providing a light flux of 535 lumens/3W at 120° viewing angle per each LED and being able to generate a tunable amplitude and length light pulses synchronized with the image captures. The CMOS sensor is a 5 Mpix rolling shutter device in ½” format, 2.2 × 2.2 µm pixel size, with a responsivity of 1.76 V/Lux-s and 5.7 × 4.28 mm active area (MT9P006 from On Semiconductors, Phoenix, AZ, USA). For the aperture definition, a set of precision pinholes 50, 200 and 500 µm) from Thorlabs (Newton, NJ, USA) has been selected. Additionally, a thin quartz cuvette (ref. 106-0.50-40, Hellma Analytics, Müllheim, Germany) has been used as fluid sample container. The cuvette is placed adjacent to the CMOS surface, making Z_2_ distance equal to the cuvette wall thickness (~1.25 mm). 

**Figure 7:**
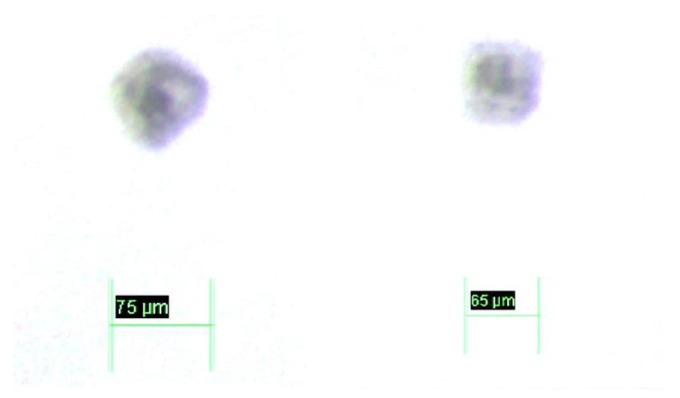
Detailed view of the patterns micro milled in the inner faces of the quartz cuvette.

Therefore, the sample volume is determined by the cuvette light path, which is 0.5 mm. Asides, the light source and pinhole have been attached to a micrometer translation stage (Thorlabs MT1/M) allowing precision Z_1_ tuning. Additionally, note that the inner walls of the cuvette walls have been micro milled with precision marks (65 μm side square and 75 μm diameter circle) that allow us to characterize the DOF, FOV and resolution of the setup. The detailed view of these micro patterns is displayed in Figure 7. 

### 2.2. Theoretical Approach

The optical requirements for the wear debris sensor have been used as the starting point for the theoretical calculations. The minimum resolution required for the detection of particles of 4 μm has been considered around 1–2 μm. The largest particle size expected should not exceed 200–400 μm. The DOF and FOV definition derives from the general request of offering the highest sampling volume possible. According to the references, in an incoherent lens-less setup, one could expect a FOV equal to the active area of the CMOS sensor, which for the current example is 5.70 mm (H) × 4.28 mm (V).

Regarding the DOF, two important considerations are needed. First, the effective DOF depends on the particle size, and different DOF are expected for 4 μm, 6 μm and >14 μm particle sizes. For instance, in the lens-based system referenced in [22], the DOF for small particles is approximately 250 μm, for medium particles it raises to 400 μm, and for large particles the system reaches almost 1 mm. Secondly, the DOF will not only be limited by the lens-less performance, but also by the light absorption happening at the sample, which according to the Lambert-Beer law, decreases logarithmically proportional to the light path length. This may generate that even at sharply focused planes a non-valid particle image may be acquired by the detector due to the lack of light intensity.

**Figure 8:**
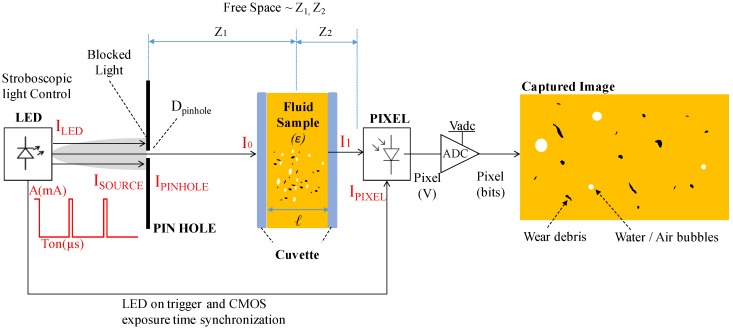
Block diagram depicting the main factors impacting in the light intensity detected at the CMOS sensor.

The already mentioned issue of the amount of light received at the CMOS needs to be further elaborated because it is a critical parameter allowing the acquisition of valid images for particle detection, especially when they are moving at relatively high velocities. As can be seen in Figure 8, this light intensity will depend on several factors as the light power emitted by the source (Lux/mA), the switch on time of the light pulse (T_ON_), the aperture diameter of the pin-hole (D_pinhole_), the free-space loses happening at Z_1_ and Z_2_ distances, the responsivity at the CMOS (V/lux-s), and of course, the light absorption occurring at the fluid sample which depends on its absorptivity (ε) and on the path length (L).

Some of these variables are defined by the state-of-the-art technology (e.g. light source efficiency, detector responsivity), whereas some others are constrained by the application requirements as the T_ON_ time, which for the target fluid flows should be sent in the range of 40 ns to 40 µs, or the path length, which for the compatible fluid range opacity, it has been set to 0.5–1 mm. These considerations will be later elaborated in Section 3.3 where the lighting system of the sensor is described.

However, parameters such as the pin-hole diameter D and Z_1_–Z_2_ distances are totally dependent on the lens-less setup selected. The following calculations are based on the work described in [39], and define the lens-less setup for meeting the requirements described above.

As mentioned earlier, for non-coherent light based lens-less setups, Z_2_ distance must be minimized. However, for the current sensor setup, there is a minimum limit for the Z_2_ defined by the sample holder structure (either a cuvette in the optical test bench or a window in the sensor prototype) is defined by the cuvette wall thickness. This holding structure needs to stand pressures of 10–15 bars, which depending on the constructive material and the area, requires to be at least 0.5 mm thick. With this assumption, and considering a sample path length of 0.5 mm:

(5) $$\begin{array}{l} Z_{2-\min}=\mathrm{sample}\;\mathrm{holder}\;\mathrm{wall}=1.25\;\mathrm{mm}\\ Z_{2-\max}=\mathrm{sample}\;\mathrm{holder}\;\mathrm{wall}+\mathrm{path}\;=1.75\;\mathrm{mm} \end{array}$$

Considering that this setup requires a fringe magnification factor, F = (Z_1_ + Z_2_)/Z_1_~1, then Z_1_ >> Z_2_, typical Z_1_ values in the literature are 2–5 cm. This relation also defines the FOV of the system, defined as FOV = Area of the sensor/F, which for F~1 remains almost equal to the full active area of the CMOS detector.

Regarding the diameter of the pin-hole, the incoherent lens-less setup requires a relatively large aperture (e.g., > 100λ–200λ). Considering the central emission peak of the white LEDs used at λ_0_ = 580 nm, we obtain: D_pinhole_ > 58 µm.

The diameter of holographic coherent diffraction (D_coh_) for each object in the focus plane is proportional to λ_0_ Z_1_/D_pinhole_. Note that for this specific case, we are targeting a direct use of the images collected, rather than needing a holographic reconstruction of the captured light intensity. Therefore, the setup will look for decreasing Z_1_ as much as possible. 

The effective width at the detector plane of each point scattered on the sample plane is defined as d_scat_ = D_pinhole_ Z_2_/Z_1_. This is relatively important for understanding the DOF and resolution performance of the proposed setup: the objects (particles) located at higher Z_2_ distances will show a higher scattering. It is clear that towards minimizing enhancing the sharpness of the images captures in the largest DOF, we should reduce D_pinhole_ and Z_2_ as much as possible.

Therefore, the choice of both the D_pinhole_ and Z_1_ is driven by a tradeoff solution for minimizing the d_scat_ and D_coh_, whereas Z_2_ must be keep the minimum possible. Additionally, for defining D_pinhole_, the impact on the light intensity filtered also needs to be taken into consideration.

Table 1 summarizes the dependencies between the lens-less parameters with the targeted optical performance indicators.

Following sections present the results obtained with different settings for D_pinhole_, Z_1_, Z_2_ and light power for imaging real lubricants (Optigear X320 Synthetic, Castrol, Berkshire, UK). Therefore, the sample volume is determined by the cuvette light path, which is 0.5 mm. Asides, the light source and pinhole have been attached to a micrometer translation stage (Thorlabs MT1/M) allowing precision Z_1_ tuning. Therefore, the sample volume is determined by the cuvette light path, which is 0.5 mm. Asides, the light source and pinhole have been attached to a micrometer translation stage (Thorlabs MT1/M) allowing precision Z_1_ tuning.) artificially contaminated with metallic particles (LS277298 Stainless Steel >45 μm AISI 316 ,GoodFellow, Huntingdon, UK).

### 2.3. Test Bench Results

**Figure 9:**
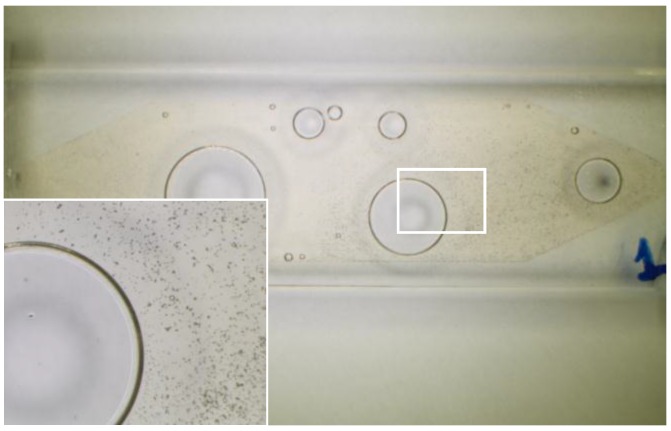
General (0.8×) and detailed view (2×) of the 0.5 mm quartz cuvette containing the contaminated lubricant sample and air bubbles.

**Figure 10:**
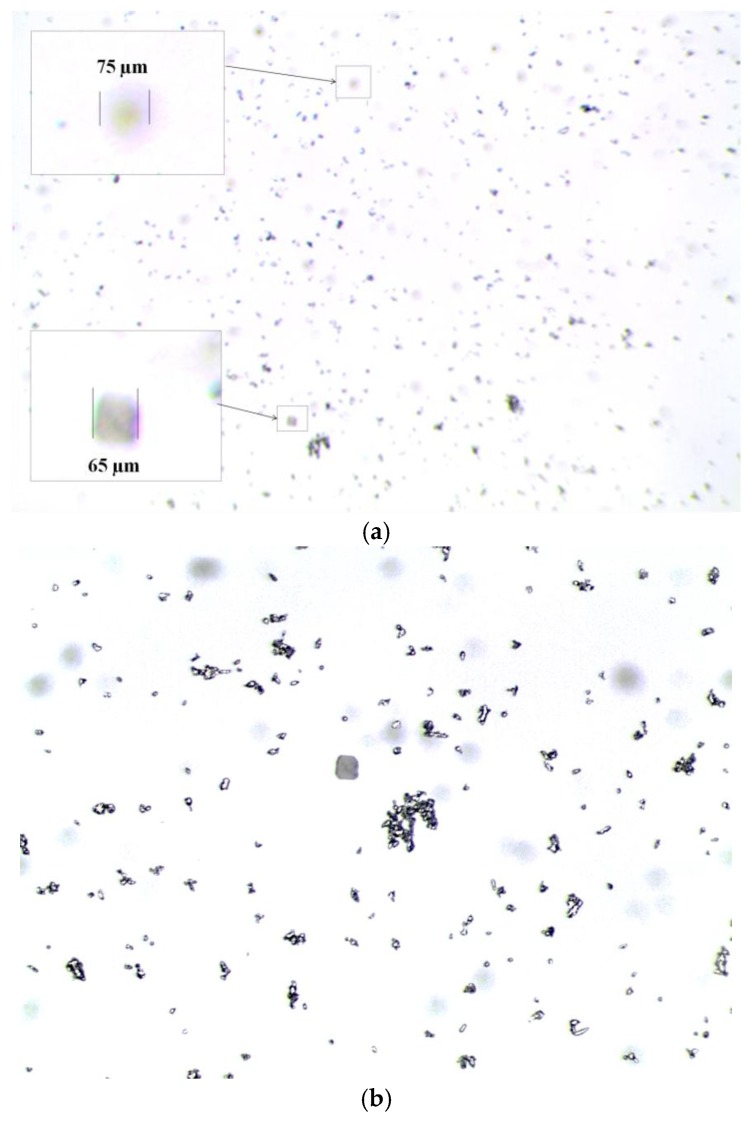
(**a**) General view (5×) and (**b**) detailed view (11.5×) of wear debris within the quartz cuvette. A detail of the micro patterns is also displayed in (**a**), where the limitation of the DOF becomes evident. In (**b**) the squared pattern could be found in the center of the image.

Figure 9 and Figure 10 show different detailed views of the cuvette sample acquired with a benchtop microscope. The presence of the wear particles and their size can be observed against the already mentioned micropatterned structures.

The cuvette has been analyzed first using the microscope optics in different magnification setups. Indeed, the level of detail achieved with the microscope observation is very high, however, the FOV and DOF is limited to the properties of regular optics. For instance, in Figure 10a the limitation in DOF is evident: when using a 5× magnification, the FOV achieved is 5485 μm by 4100 μm, but the performance in terms of DOF is very poor, as the system is almost unable to focus the two micromilled patterns that are 500 µm deep far from each other.

**Figure 11:**
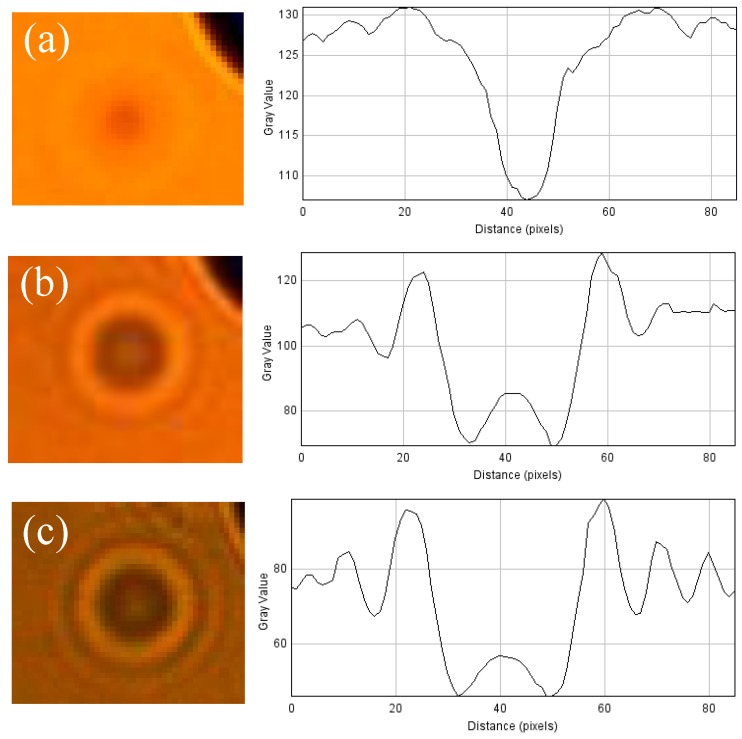
Examples of the different diffraction patterns and their linear intensity profile for different pinholes (**a**) D_pinhole_ = 500 μm; (**b**) D_pinhole_ = 200 μm; and (**c**) D_pinhole_ = 50 μm all of them with Z_1_ = 11 mm and Z_2_ = 1 mm.

**Figure 12:**
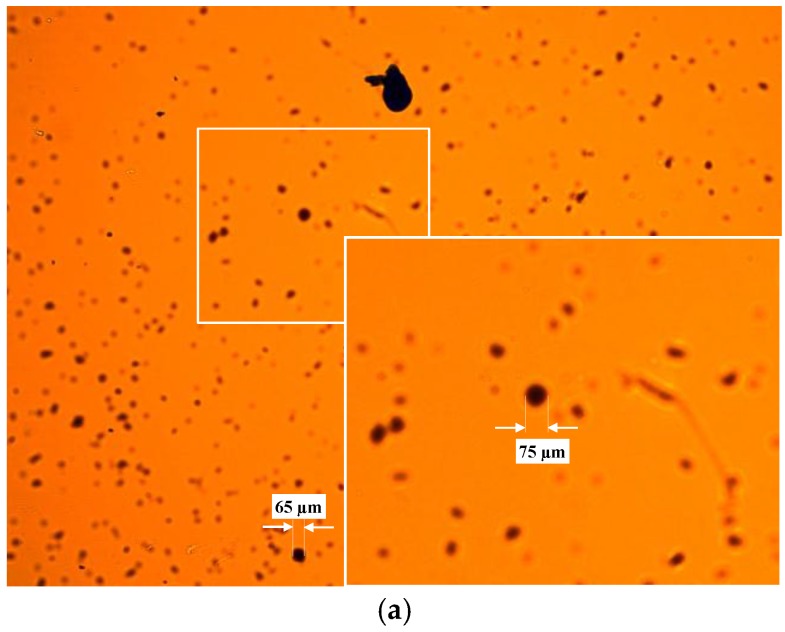

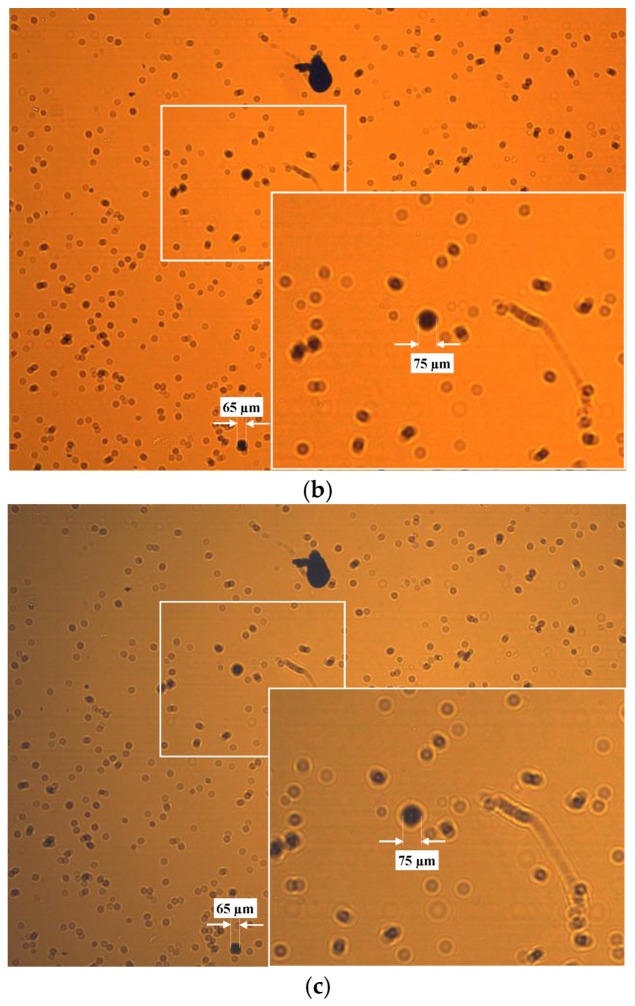
Examples of results with the lens-less test bench. Full FOV and Detailed view of a contaminated fluid sample with (**a**) D_pinhole_ = 500 μm; (**b**) D_pinhole_ = 200 μm; and (**c**) D_pinhole_ = 50 μm all of them with Z_1_ = 11 mm and Z_2_ = 1 mm. Light Power and T_ON_ pulses have been configured for achieving similar image intensity on all configurations.

When comparing with the images acquired with the lens-less test bench (for instance Figure 12a), both micropatterns are in the focus range, while the FOV is 4500 × 3400 μm^2^ remaining almost the same as for the microscope in 5× mode. However, the quality and resolution of the images acquired with the lens-less setup are affected by the diffraction patterns generated at each particle, which are inversely proportional to the pinhole diameter as described by Fraunhofer diffraction pattern for a circular aperture [40]. Figure 11 displays the diffraction patterns generated with the different pinholes.

Note that the objective of the proposed sensor system is to work with direct imaging, avoiding the need of reconstructing holograms. For this specific case, the diffraction patterns are considered as a noise source, and therefore, the design specification should work towards mitigating their presence. The following images show the result of inspecting the same fluid sample under different pinhole configurations. Additionally, even if in this case the samples were static, a stroboscopic illumination system has been used, with pulses durations ranging from T_ON_ 500 ns to 5 μs and amplitude intensities from 2 to 6 A. The settings that displayed the best performance were based on the biggest pinhole diameter, Z_1_ around 10–11 mm and the smallest Z_2_ possible, in this case approximately 1 mm and limited by the cuvette wall. 

## 3. Sensor Design

The results accomplished during the experiments at the optical test bench were considered promising enough for launching the design of the proof-of-concept sensor prototype.

### 3.1. Sensor Description

**Figure 13:**
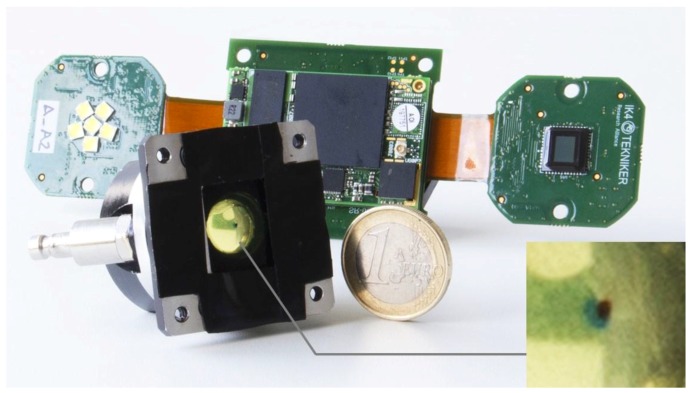
Sensor Prototype. The micromechanical sensor body is in the front, where the measurement cell is detailed. The electronic subsystem with the processor, the CMOS and the LED lighting is in the background.

The sensor solution integrates different building blocks, including micromechanics, microfluidics, photonics and electronic subsystems (see Figure 13). The micro mechanical solution includes the hydraulic connections, microfluidic sample cell, deals with positioning of optical and electronic components and additionally solves the external enclosure. The photonic subsystem integrates the CMOS camera, stroboscopic lighting, pinhole, light diffuser and two transparent glass disks to confine the fluid within the sampling cell. Besides, the sensor includes a custom embedded electronics for video acquisition, lighting control and execution of the machine vision algorithms for object recognition. The following sections describe the most challenging subsystem of the sensor: the mechanics, optics and the lighting system.

### 3.2. Mechanics and Optical Design

**Figure 14:**
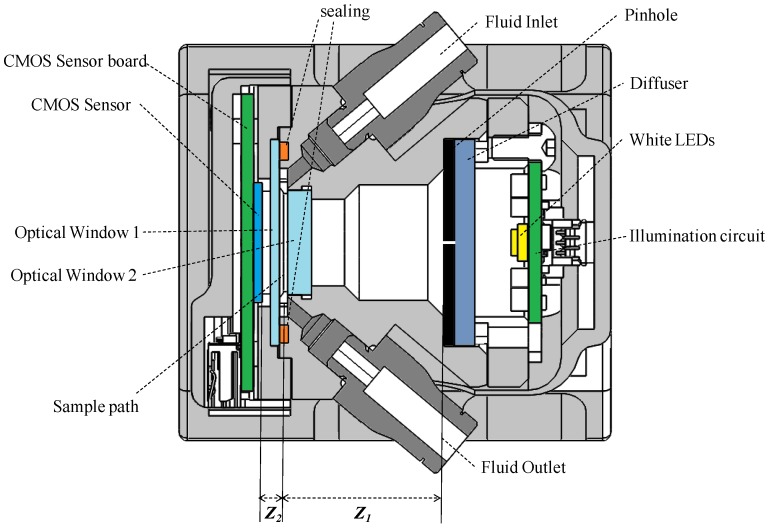
Block Diagram depicting the sensor system.

The conclusions regarding FOV, Z_1_, Z_2_ and D_pinhole_ dimensions were fed into the specifications for mechanical design of the sensor body. Matching these micro-scale requirements with relatively challenging pressures (e.g., 10–15 bar), flows (e.g., 1–3 liter/min), viscosities (e.g., 320–480 cSt), etc. requires a careful system design. For instance, the requirement for using standardized hydraulic fast plug connectors (BSP Gas 1/8) in combination with the design target of reducing Z_2_ as much as possible (~0.5 mm) has required to introduce crosswise sample inlet and outlets, due to space restrictions, as it can be observed in Figure 14.

Integrating the electronics, mechanics and optics, the sensor dimensions are approximately 35 × 45 × 45 mm, which could be considered as a very compact solution. The sensor body is fabricated in anodized aluminum and the sealing materials are fluorocarbons (for mineral or synthetic lubricant fluids) or EPDM (for measuring phosphate ester-based hydraulic fluids). Regarding the optical window closest to the CMOS sensor, which defines the Z_2_ distance, it should be keep the thinnest possible while standing the fluid pressure specification. The following formula describes the minimum thickness of glass disk before it breaks under a given pressure:

(6) $$T\left(\mathrm{inch}\right)=\;\sqrt{\frac{\mathrm{Pmax}\left(\mathrm{psi}\right)\times A\left(\mathrm{sq}.\mathrm{inch}\right)\times F}{3.12\times M}}$$

Whereas, T represents the minimum thickness in inches, A defines the unsupported area in sq/inches, P is the maximum pressure in psi, M defines the modulus of rupture (in psi) and F is a safety factor that normally is set to 7. Therefore, depending on the type of glass, different thickness could be used for tolerating a specified maximum pressures of 10 bars. In the current proof of concept, two different glass materials and thicknesses have been used. The first option is a 0.2 mm ultra-thin BK7 (M ~2400 psi) window from Edmund Optics (Barrington, NJ, USA), that have been selected with the aim of demonstrating the best optical performance as it allows a high reduction in the Z_2_ distance. Unfortunately, according to Equation (6), this BK7 option only bears 0.01 bars, meaning that its use is restricted to low pressure applications. The second candidate is a 1.1mm thick Gorilla^®^ Glass (M ~100,000 psi) disk, which allows working up to 11 bars but sets Z_2_ a little bit further, but still in the range of operation for incoherent lens-less applications. The second optical window (the one closest to the pinhole) has been resolved with a thicker BK7 glass disk (3 mm). The sample path is 0.5 mm, equivalent to tests performed with the cuvettes.

Additionally, in order to protect the CMOS sensor from the bending occurring at the glass window, a small security air gap of 0.2 mm has been defined. Therefore, the system is able to offer a minimum Z_2_ of 0.4 mm mounting the ultrathin glass disk and a maximum of Z_2_ 1.3 mm when using the Gorilla^®^ glass. Implementing the Z_1_ distance is much straightforward, and the sensor integrates a mechanical solution for holding the pinhole plate at 10 mm from the center of the sample path.

### 3.3. Light System Design

Pinhole selection and the light emitter design has been driven by the light power requirements for properly illuminating flowing lubricants. As it has been explained in earlier sections, due to the instant velocity of the particulate flowing within the lubricant, a stroboscopic lighting system is required for avoiding the generation of distorted images. In this context, the duration of these pulses is defined by the expected velocity of the objects (particles) suspended in the fluid under supervision when they go through the focusing area of the CMOS. Indeed, the duration of the illumination pulse is inversely proportional to the maximum velocity of the moving objects.

**Figure 15:**
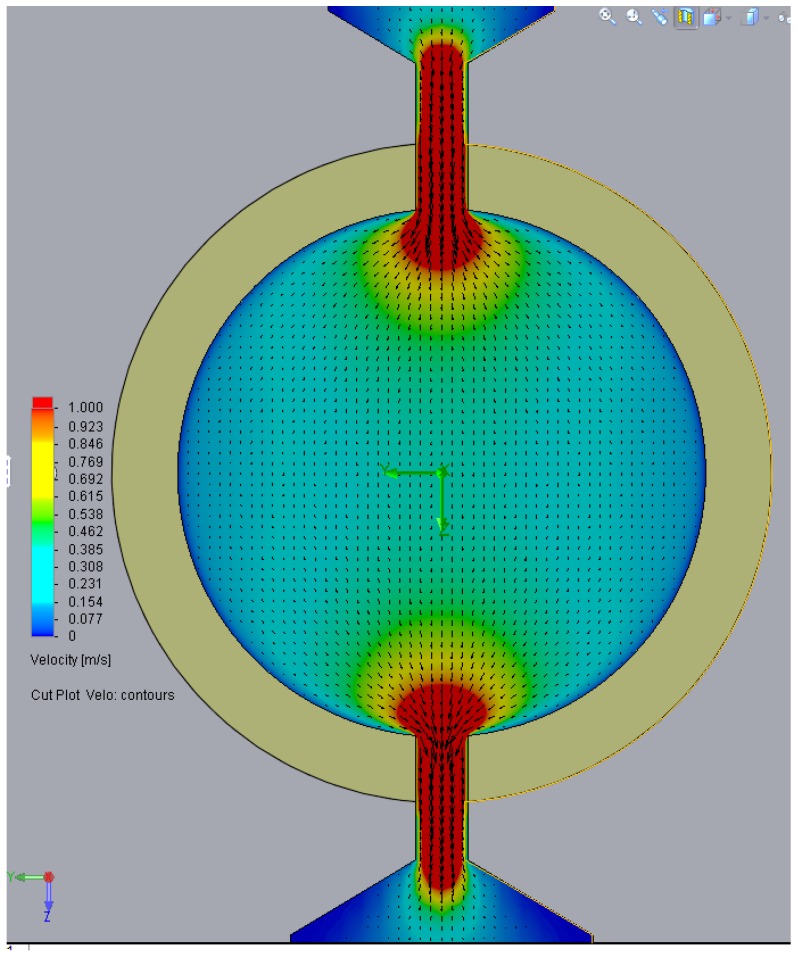
CFD simulation of sensor fluidic structure displaying the flowing velocity of the lubricant normalized to 1 m/s.

According to the Computational Fluid Dynamics (CFD) simulation displayed in Figure 15, considering a range of working pressures of 2, 5 and 10 bars, the expected laminar flow speeds of the lubricant across the microfluidic structure of the sensor are 3, 11 and 22 m/s, respectively. Therefore, the particulate suspended in the fluid will also move at similar velocities (not considering turbulence effects, etc.).

**Figure 16:**
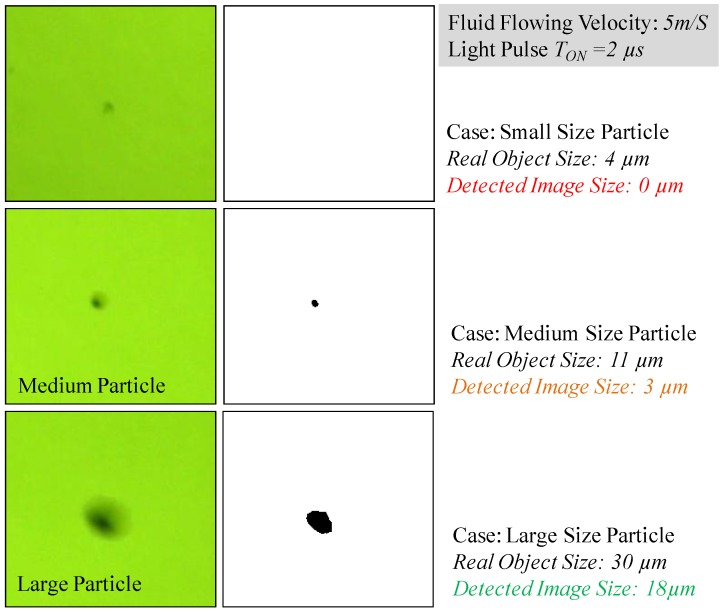
Impact of the object distortion (images on the right column) on its recognition through machine vision depending on its original size (images on the left column) for a given flowing and illumination conditions.

However, even if all particles are moving at the same velocity, the effect of the image capture distortion does not affect large and small particles alike. Large particles, even with a small distortion, will be detectable by the machine vision algorithms and there is no significant impact, being the real particle size very close to the size of the object detected. However, as the particle size gets smaller, the effects of the distortion are more pronounced, impacting both on the size and on its apparent shape, even making the smallest particles non-perceptible for the detection algorithms, as it can be observed in the example displayed in Figure 16.

In this situation, a criterion has been defined to determine which percentage of distortion generates a fatal impact for the particle detection. Considering for example that distortion will cause a bad detection if 50% of the area of the object is affected, the Table 2 shows the maximum duration of the stroboscopic light pulse for different object sizes (largest dimension) and velocity of the fluid, calculated as:

(7) Max. Acceptable Distortion (µm)=Particle Size (µm) ×0.5

(8) $$\mathrm{Max}.\;\mathrm{Acceptable}\;\mathrm{Time}\;\mathrm{Lapse}\left(µs\right)\;=\frac{\mathrm{Max}.\;\mathrm{Acceptable}\;\mathrm{Distortion}\;\left(µm\right)}{\mathrm{Flow}\;\mathrm{Velocity}\;\left(\frac{m}{s}\right)}$$

The time lapse limits the interval of time that a particle of a certain size could be moving without generating a distortion that would impact on its later recognition. Therefore, this interval defines the maximum allowed pulse duration, T_ON_, for the stroboscopic lighting system. 

With such a little time (e.g., 500 ns < T_ON_ < 4 μs) for illuminating the fluid sample, it is straightforward to conclude that a high-power light source will be required for generating a decent signal level at the detector; moreover considering the current responsivity or sensitivity of industrial CMOS with 2.2 μm^2^ pixel sizes is approximately 1 to 2 V/Lux-s (e.g., MT9P006 sensor features 1.76 V/Lux-s and the AR0330 1.9 V/Lux-s at λ = 550 nm). If generating a mid-scale intensity value at each pixel of the CMOS is set as the design objective, the light intensity budget calculation across the system could be approximately described with the following equations and considerations:

(9) $$V_{\mathrm{PIXEL}\;\mathrm{mid}-\mathrm{scale}}=0.5\times V_{\mathrm{ADC}-\mathrm{PIXEL}}=0.5\;\times 2.4\;V=1.4\;V$$

(10) $$V_{\mathrm{PIXEL}\;\mathrm{mid}-\mathrm{scale}}=R\left(\frac{V}{\mathrm{Lux}\cdot s}\right)\times I_{\mathrm{PIXEL}\;\mathrm{mid}-\mathrm{scale}}\left(\mathrm{Lux}\right)\times T_{\mathrm{ON}}\left(s\right)$$

(11) $$1.4\;V=1.76\left(\frac{V}{\mathrm{Lux}\cdot \sec}\right)\times I_{\mathrm{PIXEL}\;\mathrm{mid}-\mathrm{scale}}\left(\mathrm{Lux}\right)4\cdot 10^{-6}\left(s\right)$$

(12) $$I_{\mathrm{PIXEL}\;\mathrm{mid}-\mathrm{scale}}=0.2\cdot 10^{6}\left(\mathrm{Lux}\right)$$

Therefore, sufficient light energy needs to be provided by the system to allow that light flux getting to each pixel. The diagram depicted in the Figure 8, describes the different main considerations for the light power budget required for the current application as the light absorption happening at the sample, free space losses and the light filtering occurring at the pinhole. 

Considering that the absorptions across the free space (FSPL) and at the cuvette walls are both negligible, then, the most important factors for the light intensity losses are the absorption happening at the fluid sample and the light filtering at the pinhole. Friss Formula, which describes free space power losses as FSPL = (4π × D/λ)^2^, allows us to demonstrate the assumption of FSPL~0, being D~Z_1_~0.01 m or D~Z_2_~0.001 m. In addition, the high transmission of the quartz (above %90) across the visible light spectrum [41], demonstrates the low impact of the glass disks in the light intensity budget. Therefore, we assume that I_pinhole_~I_0_ and I_1_~I_PIXEL_*.*

The absorption of the light crossing through the sample fluid is defined by the Lambert-Beer law, which describes an exponential relation between the light entering the sample (I_0_) and the light getting across it (I_1_) as I_1_ = I_0_ × 10 ^− *εℓc*^ = I_0_ × 10 ^−*A*^, whereas *ε* is the attenuation coefficient; *c* is the amount concentration of the absorbent; and *ℓ* is the path length of the beam of light through the fluid sample; and their product represents the absorptivity (*A*) happening at the sample. *A* value of *A* = 0.5 is taken as an average absorptivity of used lubricant oil at ʎ_0_ = 580 nm for 0.5 mm path length [42,43], accordingly, the incoming light power will be lowered in a factor of: 10^−A^ = 10^−0.5^ = 0.316. Therefore:

(13) $$I_{\mathrm{PIXEL}\;}=0.316\times I_{\mathrm{pinhole}}\left(\mathrm{Lux}\right)$$

and from Equation (12):

(14) $$I_{\mathrm{pinhole}\;\mathrm{mid}-\mathrm{scale}}\;=\frac{I_{\mathrm{PIXEL}\;\mathrm{mid}-\mathrm{scale}}\left(\mathrm{Lux}\right)}{0.316}=\;0.633\cdot 10^{6}\left(\mathrm{Lux}\right)$$

At this point, the I_LED_ to I_pinhole_ relation needs to be defined to calculate the minimum light power that needs to be generated by the illumination solution to deliver an image on the camera sensor with decent brightness. As the aperture could be considered as an spatial filter that blocks a significant portion of the emitted light flux, it is straightforward to correlate the light power transmitted through the pinhole with the area of the aperture (I_pinhole_∝ D_pinhole_).

**Figure 17:**
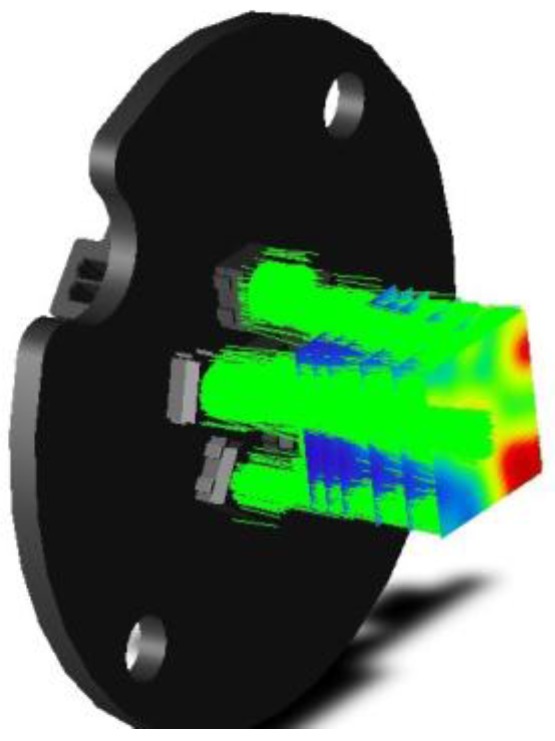
Output example of the ZEMAX simulation of the light source.

Based on the output from ZEMAX simulation (see Figure 17), a set of six LEDs have been validated for illuminating the pinhole from a distance of ~5 mm within a polished aluminum case. The white LEDs chosen offer a light flux of 178 lumens/W (9 W maximum) at 120° viewing angle. This means, that at a 5 mm distance, the illumination system, per each LED, is able to generate approximately 2,266,000 Lux/W spread following a Gaussian distribution in an area of 235 mm^2^ right before the pinhole. However, only a minimal proportion of this light flux will get across the aperture, and could be described as:

(15) $$I_{\mathrm{source}}=2,260,000\left(\frac{\mathrm{LUX}}{W}\right)\cdot \frac{1}{235{\;\mathrm{mm}}^{2}}\cdot W_{\mathrm{LED}}\left(W\right)$$

(16) $$I_{\mathrm{pinhole}}=I_{\mathrm{source}}\times A_{\mathrm{pinhole}}=2,260,000\left(\frac{\mathrm{LUX}}{W}\right)\cdot \frac{1}{235{\;\mathrm{mm}}^{2}}\cdot W_{\mathrm{LED}}\left(W\right).\pi \cdot \frac{D_{\mathrm{pinhole}}({\mathrm{mm}}^{2})}{2}$$

Combining Equation (16) with Equation (13), I_PIXEL_ is defined in dependence with the polarization of the LED and with the diameter of the pinhole:

(17) $$I_{\mathrm{PIXEL}\;}=0.316\times I_{\mathrm{pinhole}}=47,000\left(\frac{\mathrm{LUX}}{W\;.{\mathrm{mm}}^{2}}\right)\cdot D_{\mathrm{pinhole}}({\mathrm{mm}}^{2})\cdot {\;W}_{\mathrm{LED}}\left(W\right)$$

Then, for each of candidate pinholes, different LED number and polarization currents are needed for achieving the targeted mid-scale intensity level of I_PIXEL_ = $0.2\times 10^{6}$ Lux. If the saturation current is reached, and considering a fixed number of LEDs (a circular array of 6 LEDs have been arranged for the current setting), we would be forced to increase the stroboscopic pulse time above the cited 4 µs.

As mentioned, due to the pinhole filtering, only a small proportion of this light beam power will be transmitted to the sample. Table 3 summarizes the expected light power at the pixel plane considering all the assumptions and design parameters described so far. The data displayed concludes that the only feasible pinhole diameter is the 500 μm for the target fluid absorptivity and fluid flowing velocity, because it is the only setting that enables achieving the requested design objective of I_PIXEL_ of $0.2\times 10^{6}$.

**Figure 18:**
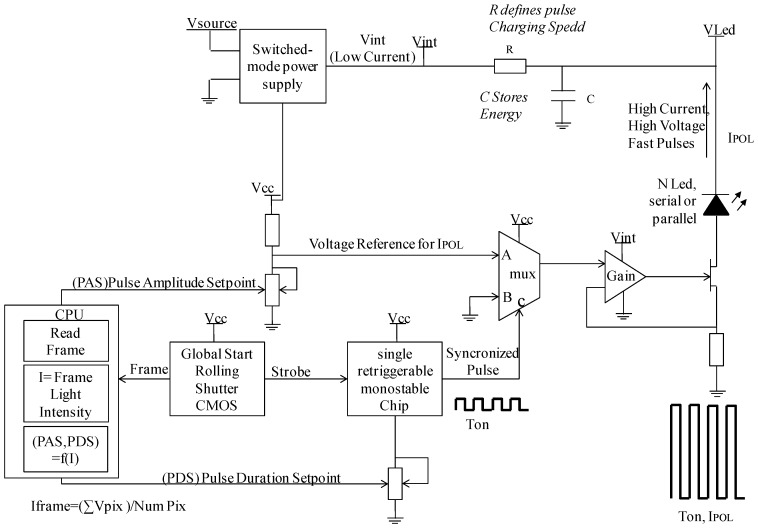
Block diagram of the stroboscopic light source of the sensor. Including the pulse length and amplitude control and the synchronization (strobe) control from the CMOS for triggering the pulse start.

A custom circuit has been designed for controlling the pulse length and intensity of the LEDs using an algorithm that is executed on the CPU. The activation time (T_ON_) and polarization current (I_0_) of the LEDs for achieving the frame intensity set point is computed for every new frame. As depicted in the Figure 18, a capacitors array is used for storing the energy required for driving the LEDs. This setup allows meeting the requirement of a very fast (T_RISE_ < 10% T_ON_) pulse switching times even for very short pulses, which is a critical design challenge, as described in [44,45].

## 4. Results and Discussion

This section describes the optical performance achieved with the sensor prototype sampling real used lubricant samples. Additionally, the performance of the machine vision algorithms is evaluated in terms of particle counting capabilities and algorithm execution time on different embedded CPU architectures.

### 4.1. Optical Results

**Figure 19:**
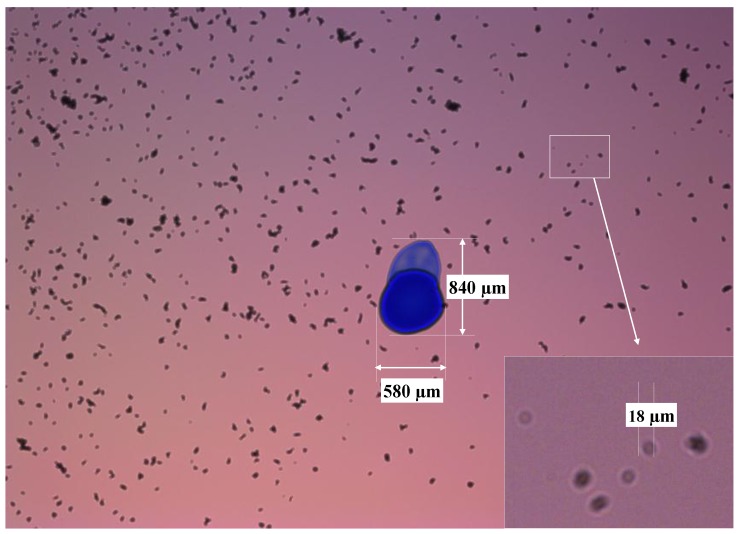
Sample image acquired with the sensor mounting the BK7 glass disk of 0.2 mm.

**Figure 20:**
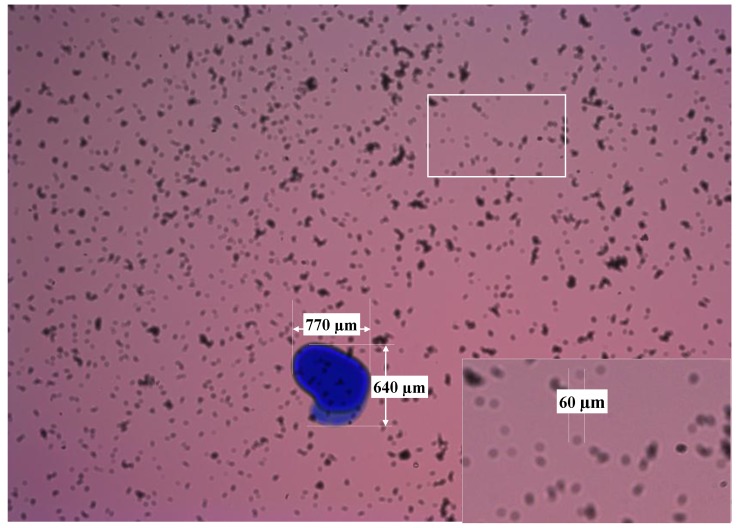
Sample image acquired with the sensor mounting the Gorilla glass disk of 1.1 mm.

The objective of the first test was determining the particle detection limit in the sensor, the FOV and the DOF. For this purpose, the glass disks were marked with ink in their faces in contact with the fluid, being these marks previously measured in the microscope as reference. Then, sensor was assembled with these disk and particles were fed into the measurement cell. Sample images of this experiment are found in Figure 19 and Figure 20.

**Figure 21:**
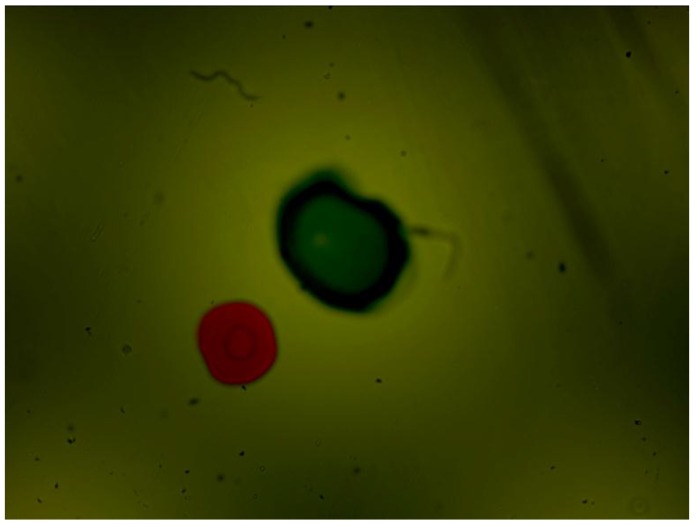
In this case the sensor mounts the Gorilla^®^ glass. Sample is Castrol Optigear (opaque sample) and the flow speed is approximately 1.0 L/min. Different particles and fibers are present in the image.

**Figure 22:**
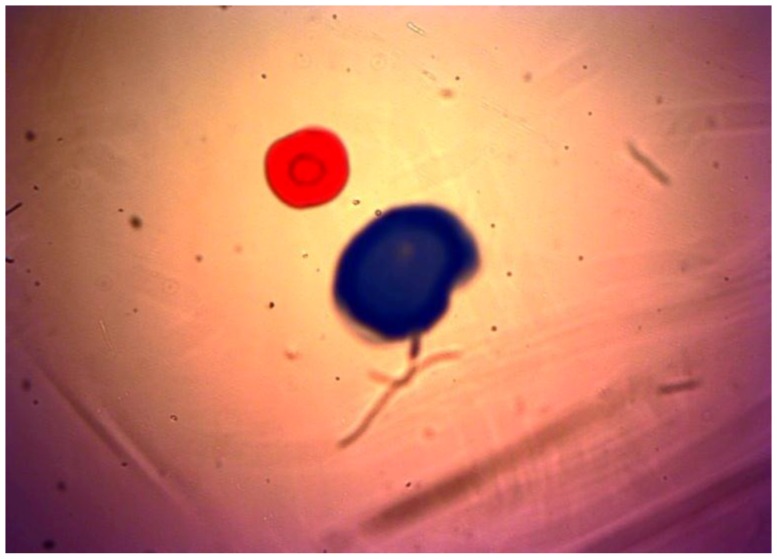
Sensor mounting the Gorilla^®^ glass disk. Sample is Texaco Meropa (medium absorptivity) and the flow speed is approximately 1.5 L/min. Asides, fibers and particles, non-homogeneity of the sample is evident, which may indicate the presence of other contaminants as varnishes.

**Figure 23:**
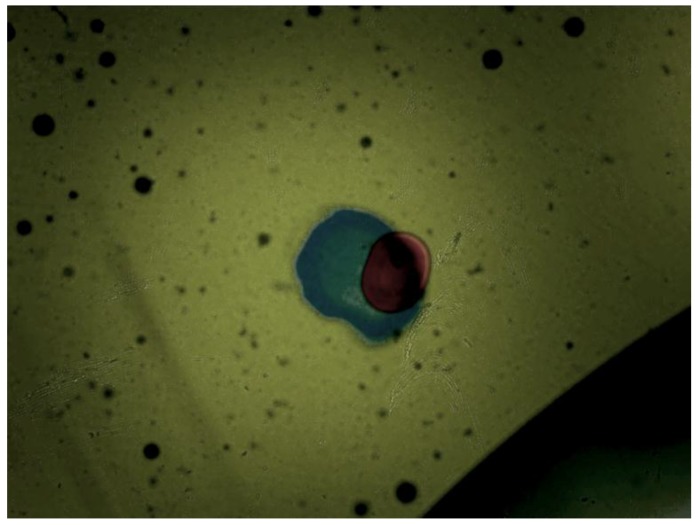
In this case the sensor is mounting the BK7 Glass disk of 0.2 mm. Sample is Castrol Optigear and the flow speed is approximately 1.0 L/min. Different particles and fibers are present in the image as well as a part of a big bubble in the bottom right corner.

**Figure 24:**
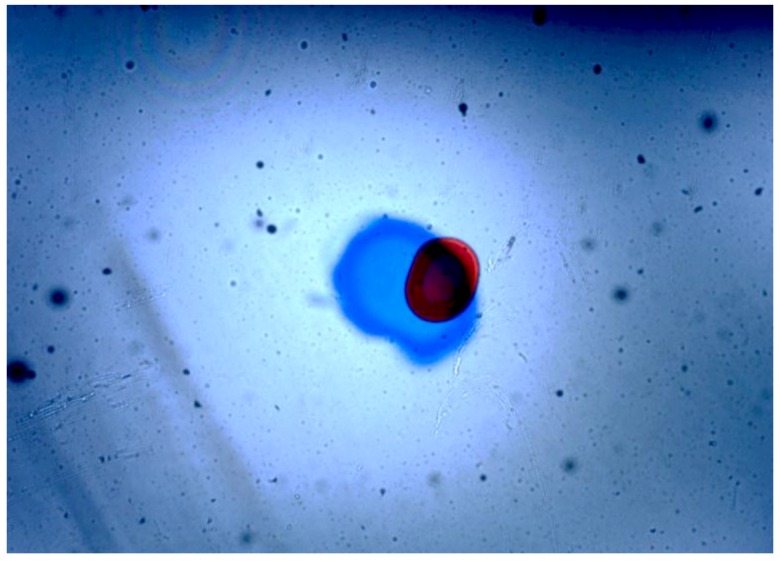
Sensor mounting the BK7 glass. Sample is Fuch Renolyn (clear fluid) and the flowing speed is approximately 1.5 L/min. Different particles and fibers are present in the image.

The detection limit was calculated using the scattering diameter for each sensor configuration, which, as described in Section 2.2, is theoretically defined as d_scat_ = D_pinhole_ Z_2_/Z_1_. The resolution has been defined calculating the μm/pix ratio using the real dimensions of the blue marks against the number of pixels. For the DOF, the glass in the face opposite to the first glass disk is also marked (see the images of the next experiment in Figure 21, Figure 22, Figure 23 and Figure 24). Therefore, introducing the values of the dimensions used in the sensor we get the following:

(18) $$d_{\mathrm{scat}\;\mathrm{GORILLA}}=D_{\mathrm{pinhole}}\frac{Z_{2}}{Z_{1}}=500\;\mu m\frac{1.1+0.2\;\mathrm{mm}}{10\;\mathrm{mm}}=65\;\mu m$$

(19) $$d_{\mathrm{scat}\;\mathrm{BK}7}=D_{\mathrm{pinhole}}\frac{Z_{2}}{Z_{1}}=500\;\mu m\frac{0.2+0.2\;\mathrm{mm}}{10\;\mathrm{mm}}=20\;\mu m$$

These theoretical values match what it is observed in the sample images. The μm/pix ratio obtained in the case of the BK7 glass disk is ~2.5 μm/pix and for the Gorilla glass is 2.43 μm/pix. The blue mark dimensions in the Gorilla^®^ glass were 770 × 640 μm and represent 307 × 282 pix in the image. On the other hand, marks in the BK7 alternative measure 580 × 840 μm and cover 238 × 357 pixel in the captured image. The FOV is calculated directly extrapolating the um/pix value to the CMOS resolution, Therefore, the FOV is covering approximately 6480 × 4860 μm^2^ due to the ~2.5 μm/pix in the 2592 × 1944 pixel matrix.

After the initial performance measurements, the sensor was plugged into the lubricant test bench. There, different fluid samples and different hydraulic settings were applied, obtaining the images displayed in the Figure 21, Figure 22, Figure 23 and Figure 24. As in the previous example, Gorilla^®^ glass and BK7 glass sensor configurations have been used and three different lubricant samples from real machines have been feed into them. These samples due to their original formulation and because of the different conditions in use (time of use, working temperatures, type of machine, mechanical stress, etc.) present different absorptivity ranges, which impacts in the frame intensity, and in the case of very opaque fluids, the sample is hardly illuminated in a homogenous way. The sample types are Renolyn (Fuchs, Staffordshire, UK), Meropa (Texaco, San Ramon, CA, USA) and Optigear Castrol and they present different contaminations (bubbles, fibers, particles, varnishes), which will be later identified through machine vision processing. Note that in this case, the sensor glass disks have been marked to help identifying the full DOF; the red ink mark is located in the first glass disk and the blue one in the second glass disk.

Table 4 summarizes a comparison between the proposed sensor, in the BK7 and Gorilla Glass configurations, with some of the most widely used commercial wear particle in-line detection systems. Notice that the sensibility to the minimum particle size of the lens-less sensor remains above the target 4 µm because no hologram reconstruction has been applied so far. According to the literature review, the use of these advanced algorithms would allow to increase the sensibility up to 1 µm as described in Section 1.2. 

### 4.2. Particle detection through Machine Vision Image Processing

After evaluating the optical performance of the sensor, in order to validate the measurement principle, particle counting algorithms should be validated in real samples monitoring. Therefore, a set of machine vision functions are executed to preprocess the image, segmentate the regions of interest, and identify and classify the objects presents on each new frame. Basically, the machine vision operations include a dynamic background compensation for eliminating the static particles or mitigating the effects of the soiling occurring at the glass disks. Then, a set of image conditioning stages are applied, such as a high pass filter and a binarization based on a variance threshold. This binary image is processed and the regions of interest are segmentated and the different features are extracted. Finally, based on these features, the bubbles and particles are separated and they are classified by their size according to the ISO standards. Halcon Embedded Machine Vision library from MVTEC Company (Munich, Germany) has been used for programming the different machine vision functions.

The execution time is a critical feature for the sensor, as it is also correlated with the ability of measuring more fluid volume per unit of time. Machine vision algorithms for high resolution images (e.g., 5 Mpix) are computationally intensive operation and, indeed, its execution speed depends on the computational power available at the embedded processor. Even if the CPU power is continuously increasing unstoppably, the results reached several ms per frame, which may be a limitation in latency critical applications. However, the current standardized structure of the embedded CPUs allows migrating the same piece of code to different processors, so the processing speed performance can be easily tested in current and future microprocessors. Therefore, depending on the real time requirements of each use case, the response time of the system could be accommodated choosing the right embedded platform.

For instance, the i.MX6 processor (Cortex A9 structured) offers the possibility of acquiring a CPU 1, 2 or 4 cores. The execution times of the algorithms have been measured in different i.MX6 CPUs and in the new family of ARM Cortex A53 devices, the Qualcomm Snapdragon 410, and are summarized in Table 5. Note that the object number within the image also impacts in the execution time, just because the decision algorithms need to be applied to a larger number of elements.

**Figure 25:**
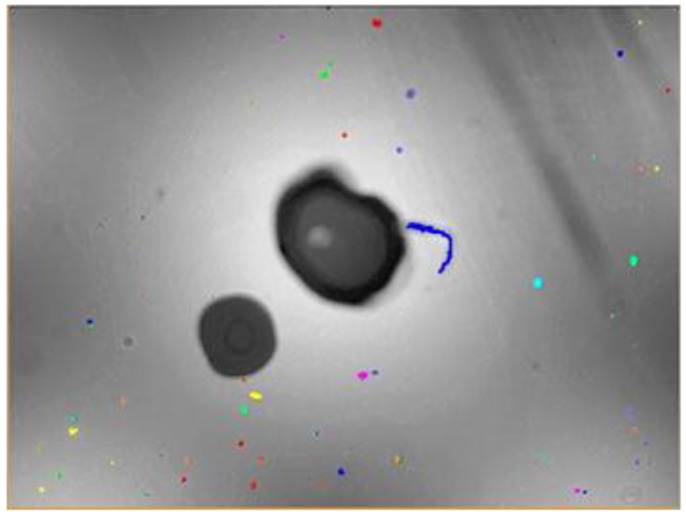
Result of processing the image of the Figure 21 with the particle detection algorithms. The processed image overlays the detected particles.

Figure 25 shows the results of applying the aforementioned algorithms to the original frame depicted in Figure 21. The particles are detected and the result is overlaid in the image. Result data outputted by the algorithms is summarized in Table 6. The absolute object measurement values are based on a prefixed parameter determining the μm/pixel proportion, which, for the example below is set to 2.5 μm/pix.

## 5. Conclusions

In this manuscript, we have presented a detailed study on the performance of a photonic micro- sensor aimed at in-line analysis of wear debris focused on the use case of industrial fluidics monitoring. The integration of lens-less microscopy and stroboscopic illumination has been accomplished to answer the challenging operation conditions in terms of sample opacity, sample flowing velocity and working fluid pressure. Specifically, we have validated the proof-of-concept analyzing the presence of wear particles in flowing lube oils based on the direct use of images acquired with a stroboscopic and incoherent lighting lens-less setup. The system settings have been optimized in a custom test-bench, achieving as preliminary results an optical performance of FOV = 5.5 mm by 4.1 mm, DOF = 500 μm (for ~70 μm objects) and 2.5 μm/pix resolution, requiring stroboscopic light pulses of about 4 μs and 6 A for dealing with fluid flow rate around 1–3 L/min. These optical settings have been transferred into the sensor design specifications, which, along with specific electronic design (including CMOS detector, stroboscopic light control, embedded CPU and communications) and the customized mechanical and micro fluidic solution, have been integrated into a compact 35 × 45 × 45 mm wear debris sensor with a really cost effective bill of materials. Additionally, examples of real lubricant samples have been described, including their particle counting and classification, as well as the execution times for the machine vision algorithms, running on reference embedded platforms such as the Cortex-A9 or the Cortex-A53. Migration from the direct use of the shadow images to the holographic reconstruction techniques for enhancing the sensor resolution towards the sub-pixel sampling has been identified as the most promising future work, as this is the way for achieving much lower detection limits for particles below 1 μm. The main advantages achieved with this proposed sensor include the cost effectiveness, compatibility with metallic and non-metallic particles, sampling volume and resolution and the potential particles shape recognition. Additionally, the compact size of the sensor allows its integration in larger hydraulic components as filters, valves, pressure sensors, etc. enabling the development of true added-value solutions in the field of industrial fluidics. Asides the industrial market, other applications, as the pharmaceutical membrane filtration systems, could benefit from the compact size and high sensibility of the proposed solution for continuously monitoring the performance of the purification and separation processes. Sampling a large fluid volume in continuous fluid flow with a decent 2D spatial resolution, this photonic micro sensor could provide a powerful tool for in-line wear debris monitoring at low resource settings.

## Figures and Tables

**Table 1:** Critical Parameters of the lens-less system.

Parameter	Design Target	Impacts on	Dependencies
D_coh_	Minimize	Resolution, DOF	∝ 1/D_pinhole_, ∝ Z_1_
I_light_	Maximize	Contrast, Flow Rate	∝ D_pinhole_, ∝ 1/Z_1_, ∝ 1/Z_2_
d_scat_	Minimize	Resolution, DOF	∝ D_pinhole_, ∝ 1/Z_1_, ∝ Z_2_

**Table 2:** Maximum Time Lapse for Illuminating a Moving Particles.

Particle Size	Small ~4 μm	Medium ~10 μm	Large ~20 μm
Maximum Acceptable Distortion (50% Blur)	2 μm	5 μm	10 μm
Flow Velocity	Maximum Acceptable Time Lapse		
	Small	Medium	Large
1.5 (m/S)	1.3 μs	3.3 μs	6.6 μs
3 (m/S)	600 ns	1.6 μs	3.3 μs
11 (m/S)	180 ns	450 ns	900 ns
22 (m/S)	90 ns	230 ns	450ns

**Table 3:** Calculations of the light budget within the sensor for different pinhole diameters and different LED polarization power. Assuming that the six LEDs are on, the different light flux intensities at the pixel are presented.

D_pinhole_	A_pinhole_	Pixel Polarization (W)				
		1 (W)	3(W)	5(W)	7(W)	9(W)
		I_PIXEL_ (Lux)				
0.5 mm	0.19 (mm^2^)	53,580	160,740	267,900	375,060	482,220
0.2 mm	0.03 (mm^2^)	8460	25,380	42,300	59,220	76,140
0.05 mm	0.001 (mm^2^)	282	846	1410	1974	2538

**Table 4:** Particle detection and classification execution time using the different CPUs architectures.

Sensor	Working Principle	Particle Type	Flow	Max. Pressure	Min. Particle	Shape Recognition
Parker FG-K19567-KW	Inductive	Only Metallic	<1.9 m/s	<20 bar	>40 μm	No
Gastops MetalScan MS4000	Inductive	Only Metallic	16.2 L/min	<35 bar	>65 μm	No
Atten2 OilWear S100	Direct Imaging	Metallic and non-metallic	Static Sample	<20 bar	>4 μm	Yes
CCJensen OCM 15	Light Extinction	Metallic and non-metallic	Static Sample	Built-in Pressure Reduction- Pumping	>4 μm	No
Lens-Less Sensor (BK7 glass)	Direct Imaging	Metallic and non-metallic	<3 m/s	<0.01 bar	>18 μm	Potential
Lens-Less Sensor (Gorilla glass)	Direct Imaging	Metallic and non-metallic	<3 m/s	<10 bar	>60 μm	Potential

**Table 5:** Particle detection and classification execution time using the different CPUs architectures.

CPU	ARM Family	Core Number	Core Bus	Core Freq.	Objects in the Image			
					0	30	68	92
					Execution Time (ms)			
i.MX6 Solo	Cortex A9	1	32 bit	1 GHz	608	865	1133	1315
i.MX6 Dual	Cortex A9	2	32 bit	1 GHz	456	648.75	849.75	986.25
i.MX6 Quad	Cortex A9	4	32 bit	1 GHz	413.44	588,2	770.44	894.2
SD 410	Cortex A53	4	64 bit	1 GHz	322.24	458.45	600.49	696.95

**Table 6:** Particle classification results according to ISO 4406 size groups for the sample image of Figure 21.

Particle Size Group	Elements Detected
>70 μm and <400 μm	1
>38 μm and <70 μm	13
>21 μm and <38 μm	29
>14 μm and <21 μm	7
>6 μm and <14 μm	22
>4 μm and <6 μm	5
